# Developing an Integrated Service Planning Tool: Lessons Learnt from Planning the WSLHD Thoracic Oncology Program

**DOI:** 10.5334/ijic.8976

**Published:** 2025-04-22

**Authors:** Kylie Smythe, David Greenfield, Anita Calderan, Paul Harnett, Alison Derrett, Adnan Nagrial, Kathy Eljiz

**Affiliations:** 1 Health Services Planning, Western Sydney Local Health District, AU; 2 School of Population Health, University of New South Wales, AU; 3 Crown Princess Mary Cancer Centre Westmead Hospital, Western Sydney Local Health District, AU; 4 Office of the Executive Director Operations, Western Sydney Local Health District, AU; 5 Crown Princess Mary Cancer Centre Westmead Hospital and Blacktown Hospital Cancer Centre, Western Sydney Local Health District, AU; 6 Health Services Research Unit, University of New South Wales, AU

**Keywords:** health service planning, strategic planning, stakeholder engagement, lung cancer

## Abstract

**Aim:**

We aim to provide practical guidelines on how to develop integrated service plans that incorporate care provided by multiple specialties.

**Introduction:**

Bringing specialties together to strategically plan future health service delivery is challenging. In Australia, collaboration between specialties is required to prepare for the introduction of the National Lung Cancer Screening Program (NLCSP). The purpose of this investigation is to provide practical guidelines on how to develop integrated service plans that incorporate care provided by multiple specialties.

**Description:**

Collaborative planning was undertaken in Western Sydney Local Health District (WSLHD) to develop a WSLHD Thoracic Oncology Program Service Plan. The planning process included oversite by a steering committee, engagement of a range of stakeholders, a series of interviews, meetings and workshops, and the documentation of the strategies and actions required to implement the plan. The planning process was analysed to produce an Integrated Service Planning Tool (ISPT).

**Discussion:**

The ISPT includes five key enablers for the planning process: foster a strong culture of collaboration; establish strategic governance; identify a patient journey framework; conduct extensive and flexible stakeholder consultation; and formalise the plan with documentation of a roadmap. Key actions for each enabler translate the ideas into activities.

**Conclusion:**

A culture of collaboration across specialties supports the development of an integrated service plan that encompasses the full patient journey. The ISPT provides a blueprint for overcoming a traditional siloed approach to service planning for diseases and conditions that require interdisciplinary care.

## Introduction

Integrated health services, as defined by the World Health Organisation, are those that provide a continuum of health care throughout the patient journey, across different levels and sites of care [1]. The benefits of integrated healthcare are multifaceted including increased patient satisfaction [2], improved patient access to services [2] and enhanced clinical outcomes [3]. While the benefits of integrated healthcare are clear, the delivery of care is often characterised by siloing of specialties [4], where groups tend to work by themselves as autonomous units within an organisation [5]. There is also siloing between the different levels of government that are responsible for healthcare [4]. In Australia the role of the national government includes developing national health policy, funding medical services and medicines and funding medical research [6]. State and Territory governments fund and manage public hospitals and the local government supports public health activities [6]. The siloing of services and tendency towards ‘refer as you go’ models of care, where individual specialists are seen as required throughout the patient journey [7], contributes to delays in diagnosis and treatment, and compromises efficiency [7]. An example of the challenge of delivering care across multiple, siloed specialty services has been identified in Australia for lung cancer care [8].

If care is to be improved, the siloing of services needs to be overcome. At a national level, service redesign needs to be undertaken with the use of tools such as logic models which define the inputs, roles and responsibilities, indicators and data sources across the whole health system [9]. At a state level, strategies and interventions for service redesign targeting population cohorts have been described as embracing intersectoral action and partnerships [10]; the goal being to develop creative, innovative solutions for clinical and non-clinical issues. At an organisational level local, efficient frontline services have been demonstrated by care models that involve continuous multidisciplinary care and clinics, consistently across the patient journey [11]. However, in many organisations, including lung cancer services in Australia, integrated care is yet to be the norm and these ideas have yet to be transferred widely into practice. For the example of lung cancer care, the need to work across specialty services applies to specialties including respiratory medicine, thoracic surgery, radiation oncology and medical oncology. There is an opportunity to redesign service delivery models for greater integration across specialties and professions (including nursing and allied health) for lung cancer and other disease and conditions that require care by multiple specialties, thereby enabling patients to move more efficiently through the stages of care [12].

Knowing how to undertake the planning steps for transforming siloed care delivery to a more integrated service model is needed [13]. There is a requirement for practical guidelines that allow organisations to navigate the planning process, supporting them to redevelop service models, and outline best practice care throughout the patient journey [14]. Grounded on this understanding, the study aim is to derive guidelines on how to develop an integrated service plan that engages services and professional specialties across the continuum of care.

Lung cancer care as a multiple specialty input is used as the setting for investigating integrated service planning. Following breast cancer, lung cancer is the second most common cancer worldwide and leading cause of cancer death [15]. In Australia, lung cancer is the leading cause of cancer death [16] and Aboriginal people are more than twice as likely to be diagnosed and die from lung cancer compared to non-Aboriginal people [17]. A critical factor in improving lung cancer outcomes is early detection [18]. However, the World Health Organisation reporting demonstrates that lung cancer is commonly diagnosed at advanced stages, limiting potential treatment options and increasing mortality [19]. Improvement is required across all stages including time to detection, time to diagnosis and the time to treatment, as these all have an impact on outcomes [20,21]. The improvement in outcomes is not only relevant to the patient but also to carers of patients [22].

## Description of the care practice

This investigation evaluated the integrated planning process that led to the development of the Western Sydney Local Health District (WSLHD) Thoracic Oncology Program Service Plan. This Plan will ensure readiness for the referrals from the National Lung Cancer Screening Program (NLCSP), commencing in Australia in July 2025 [23]. First, the WSLHD organisational context, current lung cancer care in WSLHD, the case for changing lung cancer care in WSLHD and the proposed WSLHD Thoracic Oncology Program are outlined. Then the participants in the planning process, the endorsed framework used for the plan, the step-by-step process undertaken to develop the plan and the outcome of the planning process are described.

### Western Sydney Local Health District

The delivery of public health services in the State of New South Wales (NSW), Australia is shared between the national and state governments. At the state level, the NSW Ministry of Health oversees the care that is provided by 15 local health districts [24], including WSLHD.

WSLHD provides public healthcare across more than 120 suburbs spanning 780 square kilometres. The District employs over 13,000 staff across 70 sites, including five acute hospitals and a range of integrated care and community-based services. For such a large organisation, it is essential that service planning is delivered according to a reliable framework that delivers required planning outcomes.

Western Sydney is one of the fastest growing metropolitan areas of Sydney with the population of 1.05 million people in 2021 projected to reach 1.11 million by 2026 and over 1.3 million by 2036 [25]. While WSLHD is growing it is also culturally diverse with 50% of residents born in a country other than Australia, 54% speaking a language other than English at home [26] and a high number of Aboriginal people living in the area (16,849 people in 2021) [27]. Such diversity in the population calls for service planning processes that include individual tailoring of care [28]. With such a large, diverse and rapidly growing population, it is essential that services delivered by WSLHD are efficient, effective and able to adapt to continually increasing demand [29].

### Lung cancer care in WSLHD

In the WSLHD population catchment, lung cancer represents 9% of all cancers, and the standardised incidence ratio and mortality for lung cancer is higher than the average for the state of NSW [30]. Currently, the majority of lung cancer care in WSLHD is provided at two acute hospitals, largely enabling many patients the benefit of being able to access care close to home [31,32]. Both hospitals perform greater than the recommended minimum number of lung resections per year [33] and this supports continuation of the care at two hospitals in WSLHD [33].

Lung cancer care in WSLHD may be accessed via a referral from a General Practitioner (primary care physician) to a range of specialties that commonly play a role in multidisciplinary care models. These include medical imaging, medical oncology (including chemotherapy and immunotherapy), respiratory medicine, radiation oncology (including radiation therapy) and thoracic surgery [11]. These services are available at both hospitals, while the higher tertiary level hospital also provides nuclear medicine, interventional radiology and cardiothoracic surgery services. Clinical care is provided by medical, surgical, nursing and allied health staff and a cancer care coordinator is available at each hospital.

Lung cancer care coordination is complex and resource intensive with an increase in treatment options, ageing population and incidence of comorbid conditions [34]. As is common in other countries [20,35,21], there are challenges meeting evidence based guidelines that recommend diagnosis within two weeks from referral and commencement of treatment within six weeks of referral [36]. While lung tumour boards, also known as multidisciplinary team meetings, occur weekly at both hospitals, care is otherwise delivered in a siloed approach by the required specialties. The current service model is reflective of the ‘refer as you go’ approach and also of the non-tumour based cancer care approach that is common for lung cancer care in Australia [7].

### The case for change

#### The National Lung Cancer Screening Program

The NLCSP will be introduced by the national government in Australia in July 2025 [23]. The NLCSP will enable people who are aged 55–74 years (50–74 years for Aboriginal people) [37] and meet risk assessment criteria to undergo a low dose computed tomography scan to screen for lung cancer [38]. In western Sydney the number of people in the eligible age group is projected to grow by approximately 32% from 180,806 people in 2021 to 237,886 people in 2036 [25]. Whilst the NLCSP will address the need for earlier detection of lung cancer, it does not address the challenge of improving the time to diagnosis and time to treatment. Although the initial planning of the NLCSP was completed by Cancer Australia in partnership with the Commonwealth Government Department of Health and Ageing in 2023, the plans that enable local health districts to respond to the increased number of referrals had not been developed.

The shift to more early detected cases arising from the screening program creates the opportunity for improved lung cancer outcomes for residents of WSLHD; though this is dependent on the ability of the WSLHD services to further investigate and treat the new cases. The introduction of previous screening programs, such as the National Bowel Cancer Screening Program, have been associated with significant challenges in meeting target timeframes for further investigations such as colonoscopy [39,40]. There is a risk of a similar scenario for lung cancer, if the services are not prepared for the expected increased volume of referrals that will be generated by the NLCSP.

#### Integrated service model opportunity

In considering the need to improve lung cancer outcomes and to prepare for the expected referrals generated by the NLCSP [37], WSLHD recognised the need for a more integrated approach to delivering care. This is consistent with other countries where there are also efforts to deliver care as a dedicated program for common adult tumours such as lung cancer [41]. In these programs, cancer is diagnosed and treated, and all required services from genetics and prevention, through to treatment and care of advanced disease, are available and are coordinated [41]. Examples of the programs in the United States of America include the Lung Cancer Program at Mayo Clinic [42] and the Cleveland Clinic Lung Cancer Program [43].

### The WSLHD Thoracic Oncology Program

The WSLHD Thoracic Oncology Program will integrate individual specialty services into a unified program. The program will have one point of entry, be founded upon continuous interdisciplinary care in interconnected clinics that include multiple specialties, throughout the patient journey. The integration of the services will enable seamless pathways through care, removing the common clinician and patient burden of navigating their way through complex referral processes and individual specialty scheduling arrangements [44].

The WSLHD Thoracic Oncology Program will be the first in NSW, and potentially Australia. The NLCSP will address the need for earlier detection of lung cancer across the country and the WSLHD Thoracic Oncology Program will provide the integrated, interdisciplinary approach at a local level, as recommended for high quality care [11,41]. The planning of the WSLHD Thoracic Oncology Program is, to the best of our knowledge, the first of its kind in NSW following the announcement of the NLCSP. The early detection of cases combined with the availability of efficient and effective intervention and ongoing care by a dedicated program, will place WSLHD at the forefront in improving care outcomes and patient and carer experiences.

#### Participants in the service planning process

In alignment with project governance processes that have been implemented elsewhere [45], a steering committee was established at the beginning of the planning process. The steering committee including 35 representatives from diverse specialties and services (Table 1). The categories of members included those in district executive or management roles, hospital executive or management roles, clinical lung cancer roles and external representatives from primary health care. There was equal representation from each of the two hospitals providing lung cancer care. The steering committee was responsible for developing a plan for a WSLHD Thoracic Oncology Program, including recommendations and priority actions [46].

**Table 1:** WSLHD Thoracic Oncology Program service plan steering committee and consultation participants and activities.


DETAILS	MEMBER CATEGORIES			

	DISTRICT EXECUTIVE/MANAGEMENT	HOSPITAL EXECUTIVE/MANAGEMENT	CLINICAL LUNG CANCER CARE	EXTERNAL REPRESENTATIVE

Services represented	Cancer Critical care Imaging Specialty medicine Subacute and ambulatory medicine Surgery Supportive and palliative care Allied health Multicultural health Aboriginal health Clinical innovation and redesign Population health Research and education network Health service planning	General management Medical oncology Radiation oncology Respiratory medicine Intensive care Nursing Allied health	Medical oncology Radiation oncology Thoracic surgery Respiratory medicine Nursing Dietetics Speech pathology Physiotherapy Social work Population health Aboriginal health	General Practitioner Western Sydney Primary Health Network Consumers

Number of steering committee representatives	16	10	7	2

Number of small group/individual interview participants	8	6	15	8

Total number of consultation hours	5	5.5	16	8


The steering committee oversaw the consultation process with stakeholders. The consultation with stakeholders to develop the content of the service plan involved a combination of individual and small group interviews which have been demonstrated as effective for such a process [47]. The areas examined through consultation included current service arrangements; service strengths, limitations, enablers and barriers to improved care; and opportunities for the future. The number of small group and individual interviews and consultation hours is included in Table 1.

The involvement of consumers is critical for any service redesign process [45]. While there was an aspiration for extensive consumer involvement there was also a sensitivity to the physical, mental and emotional burden carried by consumers of lung cancer services [48]. Consumers were invited to provide feedback on their experience of lung cancer services via an expression of interest that was circulated at lung cancer clinics. Individual phone interviews were a convenient and non-burdensome way for three consumers and one carer to describe their experiences and suggest areas for improvement. The consumer feedback was tabled at a steering committee meeting and incorporated in the service plan.

### Framework for the WSLHD Thoracic Oncology Service Plan

**Figure 1:**
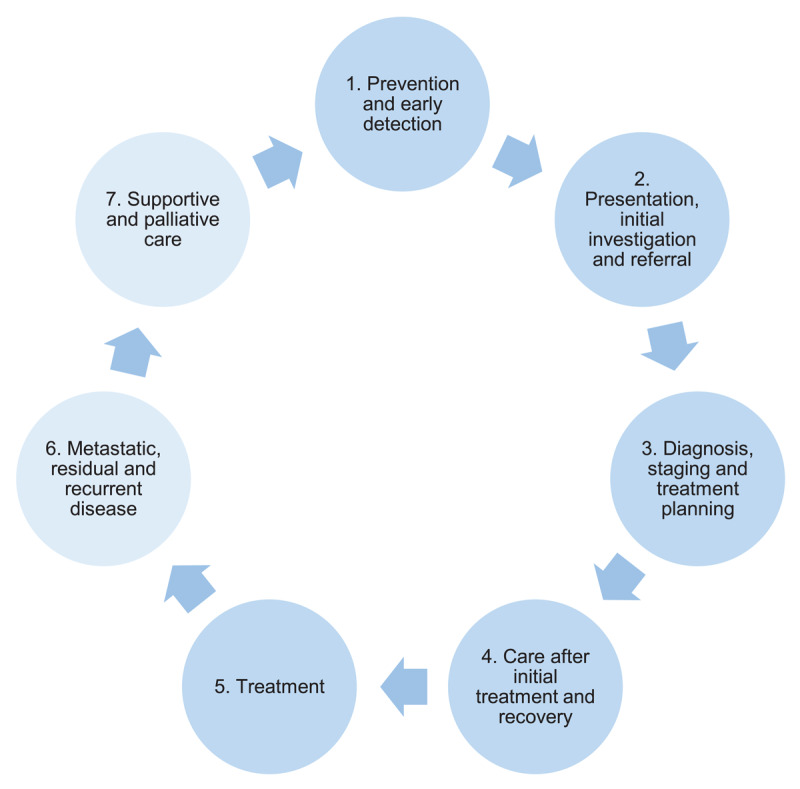
The seven stages of the Optimal care pathway for people with lung cancer [36].

The steering committee used the *Optimal care pathway for people with lung cancer* [36], to structure the planning process, ensuring that each stage of the patient journey was adequately considered. The pathway’s seven stages (Figure 1) describe key principles of care, timeframes, quality standards and support needs at each stage of the patient journey [49]. Similar pathways exist internationally, including the *National Health Service England, National Optimal Lung Cancer Pathway* [50], *Cancer Patient Pathways* in Denmark [51] and the *Tumour Specific Clinical Care Pathways* [52].

Stage 7 was originally referred to as ‘end of life care’. However people with life limiting illnesses can require care for many years [53], as is often the case for lung cancer [54], and this care is best planned and available from the beginning of the patient journey rather than just at the end [55]. Offering supportive and palliative care throughout the patient journey is standard practice in WSLHD and is recommended in the *National Health Service England, National Optimal Lung Cancer Pathway* [50]. Therefore, ‘supportive and palliative care’ was recommended as a more appropriate name for the stage.

The aim of the WSLHD Thoracic Oncology Program is to detect, treat and cure lung cancer. There is optimism that with the introduction of the NLCSP and WSLHD Thoracic Oncology Program, a greater proportion of patients will be cured of lung cancer. Following successful treatment, the patients would then move to survivorship or recovery care, including management of any mental or physical side effects of treatment.

### The WSLHD Thoracic Oncology Program service plan development process and outcome

**Figure 2:**
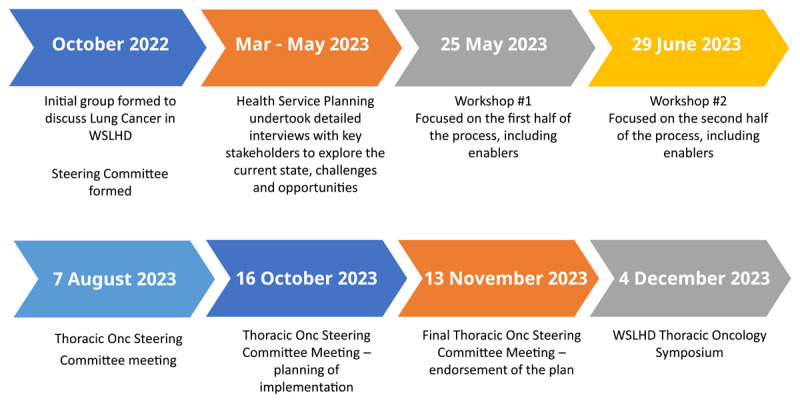
Overview of the planning process to develop the WSLHD Thoracic Oncology Program Service Plan.

The WSLHD Thoracic Oncology Program Service Plan was developed during October 2022 to December 2023 (Figure 2). The planning process began with an initial steering committee meeting and concluded with endorsement of the final plan and a WSLHD Thoracic Oncology symposium. Each step and rationale for it was determined by the steering committee in a proactive, coordinated approach (Table 2).

**Table 2:** Chronological list of steps taken to develop the WSLHD Thoracic Oncology Program Service Plan.


TIME	ACTION	WHY (RATIONALE)

October 2022	Direction from the chief executive to recommence lung cancer planning	The Chief Executive direction supported the commitment of resources (e.g. health services planning and innovation and redesign services) to the project.

	Small group meeting	The development of the service plan required regular (fortnightly/monthly) review and reflection by the small group guiding the process to ensure ongoing progress.

	Nomination of steering committee membership	A steering committee was required to provide overall leadership, direction, and responsibility for developing recommendations and priority actions [46] for the establishment of a WSLHD Thoracic Oncology Program. Membership nominees were required across specialties, across hospitals, across district executive/management roles, hospital executive/management roles, clinical roles and external roles [46].

	Circulation of a discussion paper	The discussion paper provided a way for steering committee members to develop a shared understanding of what was currently known about lung cancer care in WSLHD.

November 2022	Initial steering committee meeting	The initial meeting set the scene, confirmed the scope of the project, the consultation list and the use of the *Optimal care pathway for people with lung cancer* as a framework for the plan.

November – May 2023	Detailed consultation interviews	Detail was required from people involved in providing lung cancer care on the availability and function of current services, the challenges and the opportunities for the future.

February 2023	Steering committee meeting: consultation update	The steering committee required awareness of the issues that were being raised through consultation.

May 2023 and June 2023	Workshop 1 and 2: small group discussions about the issues identified in each step of the patient journey and each of the program enablers and development of actions to address the issues	The workshop enabled discussion across specialties and across facilities. The workshop allowed strategies and actions for the issues that had been identified to be developed collaboratively and efficiently.

July 2023	Projections: the methodology used in the planning for the National Lung Cancer Screening Program to project the number of people eligible screening, the number of people diagnosed and the number of people for each treatment pathway was applied to the local WSLHD catchment population	The plan required activity projections to inform future resource needs.

	Consumer consultation: an expression of interest was circulated at lung cancer clinics for participation in consultation. Individual phone interviews were held with three patients and one carer.	Consumer co-design is a requirement of accreditation for hospitals and ensures awareness and focus on what matters most to consumers [45].

August 2023	Steering committee meeting: tabling of consumer feedback and prioritisation of strategies and actions	Committee members required awareness of the issues raised and suggested areas for improvement from consumers [45]. Prioritisation ensured that the initial areas of focus for implementation were clear.

October 2023	Steering committee meeting: discussion about implementation working groups and a symposium	Preparation for implementation ensured that once the plan was complete, action would be taken to bring the plan to life. The symposium was proposed to communicate details of the plan to a broader audience.

November 2023	Circulation of plan	Steering committee members required the opportunity to review and comment on the detail within the plan.

	Meeting with the Chief Executive to provide an overview of the plan	The Chief Executive required a summarised version of the key features of the plan and the opportunity to ask questions.

	Revision of the plan according to feedback	Refining the plan based on feedback improved the quality and accuracy of the detail that was provided.

	Steering committee meeting: endorsement of the final plan, development of implementation committee membership list, planning for the symposium	Endorsement of the plan was a formality requested by the chairperson (Executive Director of Operations) to confirm the detail that was included in the plan.

December 2023	Chief Executive endorsement	Progressing to implementation and sharing/promoting the plan required endorsement of the plan by the CE.

	Symposium	The symposium was an opportunity to showcase the work to date and engage stakeholders outside of WSLHD.

February 2024	Publication and distribution of an abridged version of the plan	Communication about the service plan was required beyond the WSLHD Thoracic Oncology Steering Committee to include WSLHD staff and external groups such as General Practitioners, the Cancer Institute NSW and Cancer Australia. Promotion of the service plan raised awareness of the National Lung Cancer Screening Program (to be introduced in July 2025) and the preparation and planning that is taking place within WSLHD. Promotion of the service plan will also generate interest in collaborative and partnership opportunities


Documentation was recognised as critical throughout the planning process, to ensure transparency and enable communication of actions. The planning process produced 49 individual documents including a discussion paper, consultation notes, email correspondence, meeting minutes, workshop minutes and, ultimately a final draft of the WSLHD Thoracic Oncology Program Service Plan.

The planning process produced and endorsed the WSLHD Thoracic Oncology Program Service Plan. The Service Plan provides WSLHD a roadmap to achieving the optimal service delivery components that have been described in the literature including integrated, continuously multidisciplinary care [11] that is easy for patients and clinicians to navigate [56].

**Figure 3:**
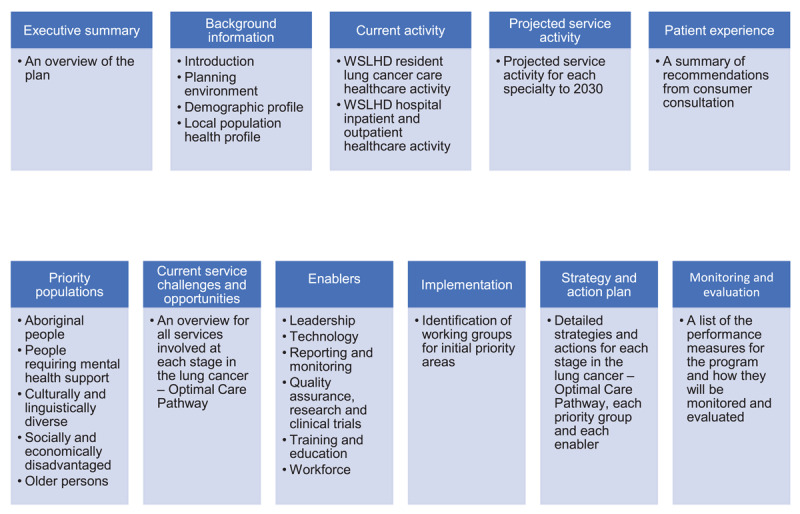
WSLHD Thoracic Oncology Program Service Plan components and content overview.

The plan includes an executive summary providing an overview, key points and a background section describing the policy, demographic and strategic context (Figure 3). Patient and carer experience information summarising the outcomes of consumer consultation is included, as are considerations for priority population groups. The strategic content includes a description of current challenges and future opportunities at each stage of the patient care pathway; the enablers necessary to support the implementation of the program; recommendations for transition into the implementation phase; a detailed execution strategy and action list; and framework for monitoring and evaluation of the program.

## Discussion

Internationally, frameworks and programs for identifying and treating, and the outcomes from lung cancer have evolved and improved significantly. The recent period has seen the gradual introduction of screening programs across the world [38] and increasing evidence for innovative models of care that integrate professionals, services and patients effectively [11].

Australia is poised to join other countries with a screening program through the introduction of the NLCSP. In response to this national initiative, WSLHD undertook a planning process to develop a Thoracic Oncology Program. The WSLHD Thoracic Oncology Program will aim to deliver integrated interdisciplinary care, consistently throughout the patient journey; the program encompasses structure and processes of best practice [11].

**Figure 4:**
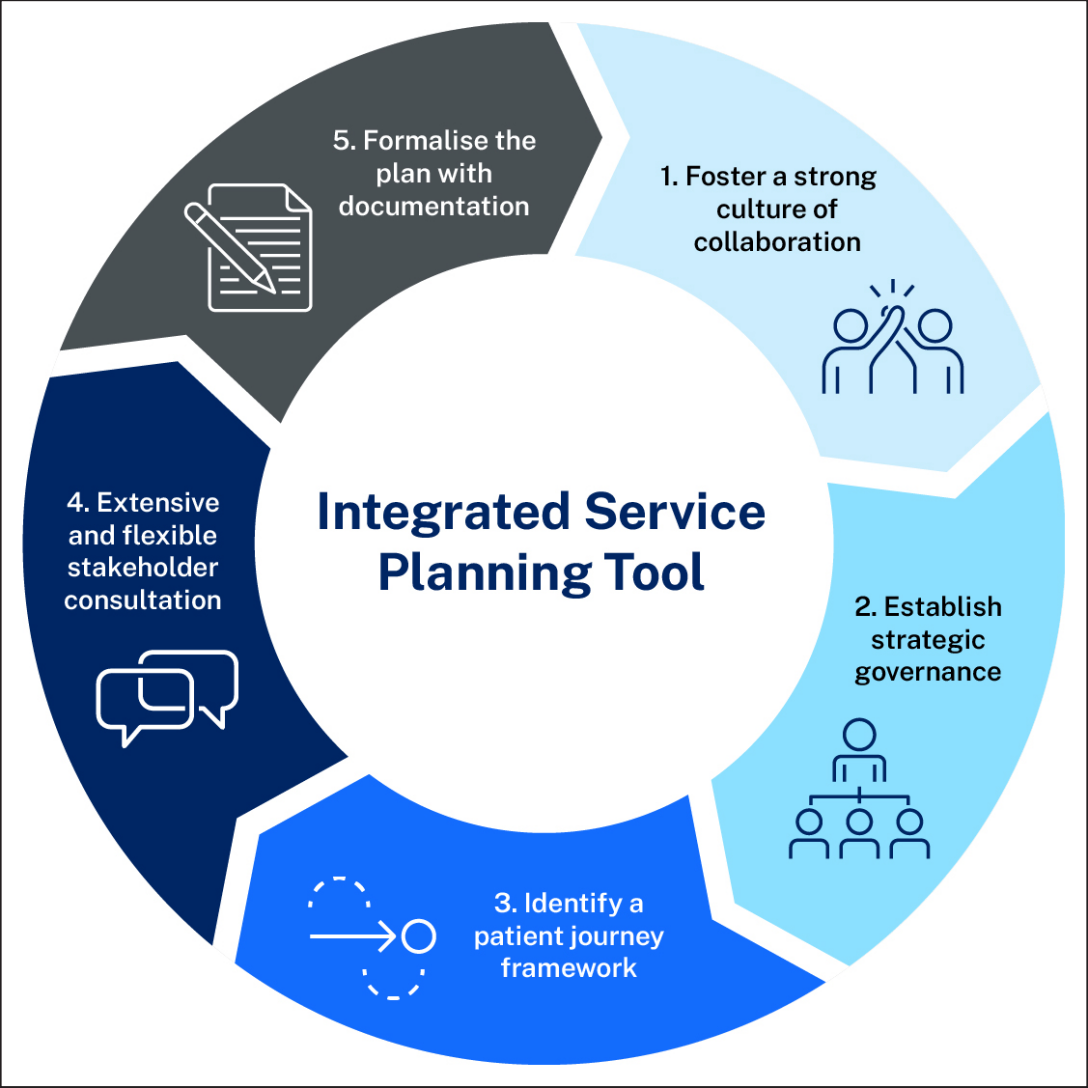
Enablers in the ISPT.

From an analysis of the planning process to develop the WSLHD Thoracic Oncology Program Plan, an Integrated Service Planning Tool (ISPT) has been derived (Figure 4). The ISPT will assist organisations who are focused on effective and efficient planning of resources and creating patient-centred services. The process described in the ISPT may be replicated to develop integrated programs that are recommended internationally [43,57] for common chronic diseases and conditions that require interdisciplinary care by multiple specialties. These include, but are not limited to, other cancers, haematological and autoimmune conditions.

An effective integrated service planning model is founded upon five key enablers: initiation of a collaborative culture which will continue through to the delivery of the integrated program [58]; establishment of strategic governance [46]; identification of a patient journey framework [49], ensuring that all steps within the care continuum are addressed [1]; the engagement of a broad range of stakeholders, using a flexible approach that ensures contributions from a range of clinical specialties, executive staff and consumers [59]; and, formalising the plan by documenting a roadmap [60].

The first enabler is fostering a strong culture of collaboration. Establishing a shared vision at a strategic level across services and professions is essential to set a platform for future work as an integrated service [58]. Opportunities for collaboration include small focus groups, meetings, workshops and email discussion threads. Symposiums that include representatives from external organisations also provide collaboration opportunities. Collective discussions about the future service promote and improve communication, relationships and trust, and redesigned care transition processes and coordination [58]. The intended outcome is a collaborative culture focused upon delivering an integrated, interprofessional service encompassing the care continuum.

The second enabler is establishment of strategic governance, with oversight by senior representatives [46]. The development of a steering committee is endorsed by the most senior executives in the organisation and clinical and executive co-chairs are nominated. The steering committee membership is formed by senior representatives from the stakeholder groups that are involved in the planning. The steering committee meetings provide a forum for all opinions to be shared and issues such as power dynamics and competing priorities are managed through the leadership of the clinical and executive co-chairs. The frequency, duration and location of meetings is discussed and confirmed at the first steering committee meeting. The responsibilities of the steering committee include setting strategic priorities and advocating for access to resources needed for innovative, safe care delivery [61]. The steering committee oversees the planning process, reviews consultation outcomes and endorses a final service plan [46].

The third enabler is identifying a patient journey framework to structure the plan. Using an endorsed care pathway in the service planning process provides a structure for engagement. This action ensures the development of safe, high-quality and evidence based services [62]. Exemplar models can be adopted or adapted as necessary; in the field of cancer, care pathways are readily available [50,51,52]. Care pathways suitable for adaption exist for other specialty areas, such as cardiology [63], respiratory disease [64] and gastroenterology [65]. The proposed patient journey framework is endorsed by the steering committee at the early stages of planning and guides the structure of the final plan that is produced.

The fourth enabler is extensive and flexible stakeholder engagement, with both equally important. Extensive stakeholder engagement, a point known in many activities, including research [66], environmental planning and healthcare delivery [67] is often simplified leading to implementation problems and conflicts [61]. Involving multiple professionals from services across the care continuum ensures that plans align with expectations, resources and capabilities; in other words, they are both credible and feasible [59]. Consumer participation, or co-design [29], ensures that key care activities impacting on patient experience and outcomes are addressed [45]. Flexibility in the approach to consultation is as vital. Stakeholders are commonly faced with time constraints and competing priorities [68]. Flexibility by planners in timing and mode of engagement can address this challenge – a working principle needs to take whatever action that has the benefit of increasing participation and overcomes barriers [69].

The fifth enabler is documentation. Documents engage, link, combine, unite, and distribute information, knowledge, and ideas; and, in doing so generate emotions, all of which contributes to the enhancement of organisational performance [70]. The service planning process generates many documents in the form of consultation notes, meeting minutes, strategy and action tables and email correspondence. This information is collated and synthesised as an initial draft which is circulated to steering committee members for review. The plan is revised according to steering committee feedback and a final plan is provided for steering committee endorsement. The final, endorsed plan provides an organising focus and blueprint for future actions [71,72].

The ISPT with related actions (Table 3) provides a practical tool for service planning with other specialties, teams and organisations. The ISPT gives a structure and process to discuss and implement change. It provides direction and is a coordinating mechanism during, what is often stressful and uncertain periods of organisational redevelopment. The ISPT may be used as a practical checklist throughout the integrated planning process with a third column added to track progress against each of the actions listed.

**Table 3:** The ISPT including five enablers and related actions.


ENABLER		ACTIONS

1.	Foster a strong culture of collaboration	Establish a shared vision amongst all involved Facilitate regular collaborative opportunities including small group discussions, meetings and workshops Host a symposium which allows collaboration with external stakeholders Circulate frequent email correspondence summarising progress on the plan and inviting feedback

2.	Establish strategic governance	Obtain senior executive endorsement to commence the planning process Identify clinical and executive co-chairs and steering committee membership Convene and minute regular, steering committee meetings to review the collated service planning information and determine an ongoing direction Obtain steering committee endorsement of the service plan

3.	Identify a patient journey framework	Review the literature for published patient care pathways and established models of care Identify a patient care pathway or model of care framework to guide the structure of the plan Apply any necessary amendments to the framework Obtain endorsement of the proposed framework by the steering committee

4.	Extensive and flexible stakeholder consultation	Include representatives at an organisational, facility and clinical service level in the service planning process Ensure that input is obtained from all relevant clinical specialties and clinical support services Obtain input from external representatives such as primary care clinicians and managers and consumers in the service planning process Allow flexible timing and location (including online options for consultation)

5.	Formalise the plan with documentation	Prepare a draft of the service plan for review by the steering committee Amend the draft according to feedback Provide a consolidated list of amendments and the rationale Provide a final draft to the steering committee for endorsement


## Lessons learned

- An integrated service planning process establishes a strong culture of collaboration from which future work may leverage.
- Establishing strategic governance arrangements that include executive and clinical representation allow oversite of the planning direction and facilitates effective decision making.
- An established optimal care pathway serves as an effective framework for structuring the planning discussions, consultation and final plan.
- Involvement of a broad range of stakeholders ensures that the plan is reflective of a wide range of views and interests; flexibility in the time and modality for input allows for effective engagement and collaboration.
- Formalising the planning process with documented service plan provides a roadmap, ensuring shared understanding of the strategy and the actions to be taken.

## Conclusion

Overcoming traditional silos and developing integrated healthcare services can be a challenge for healthcare organisations. However, interdisciplinary service models, where care is interconnected between specialties throughout the patient journey, are achievable. An integrated service planning process, as described in the ISPT, provides healthcare organisations with a practical tool on how to develop integrated services plans that incorporate care provided by multiple specialties. With a step-by-step framework, health services may face future planning tasks with an organised, structured and efficient approach. The net outcome is increased patient satisfaction, improved patient access to services, and enhanced clinical outcomes from engaged professionals.
